# Supplementary material for: Predicting visual function by interpreting a neuronal wiring diagram
Source: Nature. 2024 Oct 2;634(8032):113–23. doi: 10.1038/s41586-024-07953-5 (PMC11446822; doi:10.1038/s41586-024-07953-5)
Supplement: Supplementary file 6 — CRF and ERF predictions for individual TmY4 and TmY9 cells. Analogous to Supplementary Data 3, but for TmY target types. Shown are the top four monosynaptic pathways, the strongest pathway passing through each of the top ten intermediary types (ranking from Extended Data Fig. 7), and the trisynaptic pathway Tm1–TmY–Dm3–TmY (see the section entitled Prediction of spatial normalization). [file 41586_2024_7953_MOESM6_ESM.zip › DataS4/TmY9q/720575940607556809.pdf]

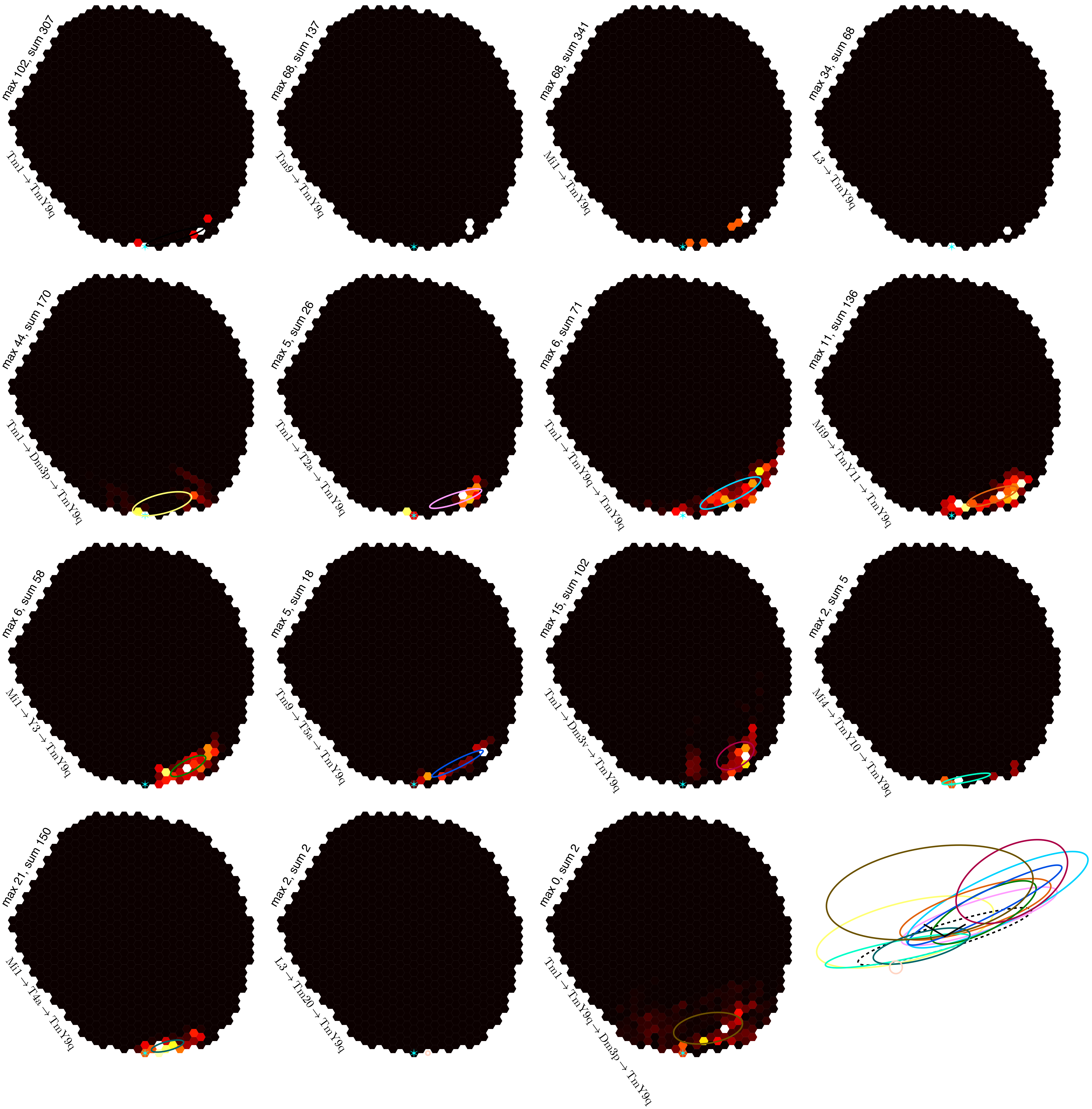

max 102, sum 307
Tm1 → TmY9q
max 68, sum 137
Tm9 → TmY9q
max 68, sum 341
Mi1 → TmY9q
max 34, sum 68
L3 → TmY9q
max 44, sum 170
Tm1 → Dm3p → TmY9q
max 5, sum 26
Tm1 → T2a → TmY9q
max 6, sum 71
Tm1 → TmY9q → TmY9q
max 11, sum 136
Mi9 → TmY11 → TmY9q
max 6, sum 58
Mi1 → Y3 → TmY9q
max 5, sum 18
Tm9 → T5a → TmY9q
max 15, sum 102
Tm1 → Dm3v → TmY9q
max 2, sum 5
Mi4 → TmY10 → TmY9q
max 21, sum 150
Mi1 → T4a → TmY9q
max 2, sum 2
L3 → Tm20 → TmY9q
max 0, sum 2
Tm1 → TmY9q → Dm3p → TmY9q
